# From secondary data to Population Data Science: remembering 40 years of scientific production within CSP pages

**DOI:** 10.1590/0102-311XEN087624

**Published:** 2024-06-21

**Authors:** Cláudia Medina Coeli

**Affiliations:** 1 Instituto de Estudos em Saúde Coletiva, Universidade Federal do Rio de Janeiro, Rio de Janeiro, Brasil.

It was a great joy accepting the invitation to write this editorial. A special opportunity to celebrate together with Marilia Sá Carvalho, Luciana Dias de Lima, Luciana Correia Alves and the entire CSP community the 40th anniversary of this important editorial project, in which I had the honor of working for nine years as Coeditor-in-Chief. Having as main research focus the development of techniques and the use of secondary databases, reviewing the scientific production of this topic within CSP allowed me to recall articles that were fundamental references for my education and development of my research projects.

The first CSP issue appeared in 1985. Internationally the sale of personal computers (PCs) ^1^ was gaining momentum, followed in the early 1990s by the opening of access to the World Wide Web (WWW) ^2^ to the public. These advances were significant for popularizing information technologies.

Administrative databases began to be used as secondary data sources in Public Health research ^3^. In the 1990s, and in the first decade of the 2000s, Data Centers were implemented in Australia, Canada, and the United Kingdom. In these organizations, administrative databases are linked continuously, and the resulting anonymized datasets can be accessed by researchers to develop their projects ^4^.

In the same period, Brazil created the Brazilian Health Informatics Department (DATASUS, acronym in Portuguese) ^5^ in 1991, which contributed significantly to accessibility to the Brazilian administrative databases. The model adopted for data dissemination was, however, different from the Data Center mentioned above. Two access modalities were made available: one through an online tabulator, which allows to create tables of the main national Health Information Systems; the other by the dissemination of unidentified microdata. The databases were initially distributed on monthly compact discs (CDs), and later made available for online downloads. Information on births, deaths, notifiable diseases, primary care, outpatient and hospital care, health facilities and public budget began to be made available not only to researchers, but also to the population at large. This Open Data model is unique for its innovation, data variety, time and territorial coverage of the databases, and inclusive access. Digital format information of interest to health care also began to be made available by different institutions such as the Brazilian Institute of Geography and Statistics (IBGE, acronym in Portuguese), the Brazilian National Supplementary Health Agency (ANS, acronym in Portuguese), the Brazilian Health Regulatory Agency (Anvisa, acronym in Portuguese), as well as state and municipal Health Departments.

Even before digital dissemination, administrative data, especially on mortality, were used in Brazil for Public Health research. However, the ease of access provided by the adhesion of Brazilian institutions to the open data model encouraged this type of use. By consulting PubMed, I identified 461 articles published in CSP that used administrative data, of which 86 addressed quality-related topics. Among the latter, the article stemming form Claudia Risso de Araujo Lima’s thesis stands out ^6^. Claudia, who was a member of the DATASUS team, was one of those responsible for implementing the health information dissemination policy in Brazil. Published in 2009, her article continues to be referenced (96 citations in the Scopus database). Her major contribution is to review quality dimensions in the evaluation of Brazilian health information systems.

Publishing articles that assess the quality of both information systems and processes for linking databases meets a growing demand for the adoption of good practices in conducting and reporting studies that use secondary data ^7^ ^,^ ^8^. An editorial ^9^ and a perspective paper ^10^ reinforce CSP’s editorial policy of promoting the responsible use of administrative databases in research.

CSP has also published four methodological articles presenting computational routines for database processing. Three solutions focused on record linkage ^11^ ^,^ ^12^ ^,^ ^13^ and the fourth, the Microdatasus ^14^ package, optimizes the download and pre-processing of microdata made available by DATASUS. In 2000, Reclink was published as a free but closed-source software ^11^. The new OpenReclink version was published in 2015 as open source ^12^. EPPD ^13^ and Microdatasus ^14^ are also open source, in compliance with CSP’s editorial policy of adhering to open science ^15^.

Information technologies saw a rapid expansion in these 40 years. Advances in the capacity to capture, process, store, communicate and analyze data occurred successively, with incremental advances in each area stimulating advances in the others. Currently, we can process large amounts of information in real time. Unstructured data in different formats, such as texts in documents or social networks, images, and sensor outputs, are new sources for secondary use in research. Moreover, techniques developed by Information Science such as data mining, machine learning, and large language models (LLMs) were introduced into health research. These innovations led to the creation of a new disciplinary field, called Population Data Science ^16^ ^,^ ^17^ which, through the organization, integration, linkage, and analysis of individual and contextual data, intends to generate population level evidence valuable for society. Articles on the development or application of record linkage techniques have been published in CSP since the 2000s. Recently, with the greater dissemination in Public Health of Information Science techniques, articles using data mining, text and machine learning have been published.

In addition to technical issues, Population Data Science seeks models for managing information access that balance the right to personal information protection with the potential benefits to society of using administrative databases in research, a topic addressed by more than one article published in CSP ^18^ ^,^ ^19^ ^,^ ^20^.

Over the course of 40 years, CSP has published articles addressing the main topics of Population Data Science, promoting good practices in the use of secondary data in research of interest to society. Consistent with its mission, it proved to be a crucial vehicle for circulating ideas and methods in this field.

## References

[1] McCracken H TIME's Machine of the Year, 30 years later..

[2] Redator Rock Content Conheça a história da Internet, sua finalidade e qual o cenário
atual..

[3] Boslaugh S (2007). Secondary data sources for public health: a practical guide.

[4] Coeli CM, Pinheiro RS, Camargo KR (2015). Conquistas e desafios para o emprego das técnicas de record
linkage na pesquisa e avaliação em saúde no Brasil.. Epidemiol Serv Saúde.

[5] Ministério da Saúde (2002). Departamento de Informática do SUS. Trajetória 1991-2002.

[6] Lima CRA, Schramm JMA, Coeli CM, Silva MEM (2009). Revisão das dimensões de qualidade dos dados e métodos aplicados
na avaliação dos sistemas de informação em saúde. Cad Saúde Pública.

[7] Leonelli S (2022). A pesquisa científica na Era do Big Data: cinco maneiras que mostram
como o Big Data prejudica a ciência, e como podemos salvá-la.

[8] Christen P, Schnell R (2023). Thirty-three myths and misconceptions about population data from
data capture and processing to linkage. Int J Popul Data Sci.

[9] Coeli CM (2015). A qualidade do linkage de dados precisa de mais
atenção. Cad Saúde Pública.

[10] Coeli CM, Pinheiro RS, Carvalho MS (2014). Neither better nor worse, simply different. Cad Saúde Pública.

[11] Camargo KR, Coeli CM (2000). Reclink: aplicativo para o relacionamento de bases de dados,
implementando o método probabilistic record linkage.. Cad Saúde Pública.

[12] Camargo KR, Coeli CM (2015). Going open source: some lessons learned from the development of
OpenRecLink.. Cad Saúde Pública.

[13] Brustulin R, Marson PG (2018). Inclusão de etapa de pós-processamento determinístico para o
aumento de performance do relacionamento (linkage)
probabilístico. Cad Saúde Pública.

[14] Saldanha RF, Bastos RR, Barcellos C (2019). Microdatasus pacote para download e pré-processamento de
microdados do Departamento de Informática do SUS (DATASUS). Cad Saúde Pública.

[15] Carvalho MS (2015). Aberto, por quê. Cad Saúde Pública.

[16] McGrail K, Jones K, Akbari A, Bennett T, Boyd A, Carinci F (2018). A position statement on population data science the science of
data about people. Int J Popul Data Sci.

[17] Coeli CM (2022). Ciência de dados populacionais. Epidemiol Serv Saúde.

[18] Ventura M (2013). Lei de acesso à informação, privacidade e a pesquisa em
saúde. Cad Saúde Pública.

[19] Ventura M, Coeli CM (2018). Para além da privacidade direito à informação na saúde, proteção
de dados pessoais e governança. Cad Saúde Pública.

[20] Keinert TMM, Cortizo CT (2018). Dimensões da privacidade das informações em saúde. Cad Saúde Pública.

