# Supplementary material for: From secondary data to Population Data Science: remembering 40 years of scientific production within CSP pages
Source: Cad Saude Publica. 2024 Jun 21;40(6):e00087624. doi: 10.1590/0102-311XEN087624 (PMC11192569; doi:10.1590/0102-311XEN087624)
Supplement: Translation

# Translation: De dados secundários à Ciência de Dados Populacionais: recordando 40 anos da produção científica nas páginas de CSP

**DOI:** 10.1590/0102-311XPT087624

**Authors:** Cláudia Medina Coeli

**Affiliations:** 1 Instituto de Estudos em Saúde Coletiva, Universidade Federal do Rio de Janeiro, Rio de Janeiro, Brasil.

Foi com grande alegria que aceitei o convite para escrever este editorial. Uma oportunidade especial para celebrar junto a Marilia Sá Carvalho, Luciana Dias de Lima, Luciana Correia Alves e toda a comunidade de CSP os 40 anos deste importante projeto editorial, no qual tive a honra de atuar por nove anos como Coeditora-Chefe. Tendo como meu foco principal em pesquisa o desenvolvimento de técnicas e o uso de bases de dados secundários, rever a produção científica desse tema em CSP me permitiu recordar artigos que foram referências fundamentais para minha formação e desenvolvimento de meus projetos de pesquisa.

O primeiro número de CSP nasceu em 1985. No contexto internacional, a venda de computadores pessoais (PC) ^1^ ganhava momento, sendo seguida, no início dos anos 1990, pela abertura ao público do acesso à World Wide Web (WWW) ^2^. Esses avanços foram significativos para a popularização das tecnologias de informação.

Bases administrativas passaram a ser empregadas como fontes de dados secundários na pesquisa em Saúde Coletiva ^3^. Nos anos 1990 e na primeira década dos anos 2000, Centros de Dados foram implantados na Austrália, Canadá e Reino Unido. Nessas organizações, bases administrativas são vinculadas continuamente, e os conjuntos de dados anonimizados resultantes podem ser acessados por pesquisadores para o desenvolvimento de seus projetos ^4^.

No Brasil, foi criado, em 1991, o Departamento de Informática do Sistema Único de Saúde (DATASUS) ^5^, que contribuiu significativamente para a acessibilidade às bases administrativas brasileiras. O modelo adotado para a disseminação de dados foi, contudo, diferente do Centro de Dados mencionado anteriormente. Foram disponibilizadas duas modalidades de acesso. A primeira é por meio de um tabulador *online* que permite criar tabelas dos principais sistemas de informações em saúde nacionais. A segunda consiste na disseminação de microdados não-identificados. Inicialmente, as bases eram distribuídas em *compact discs* (CDs) mensais, sendo, posteriormente, disponibilizadas para transferências *online*. Informações sobre nascimentos, óbitos, doenças e agravos de notificação, atenção básica, cuidados ambulatoriais e hospitalares, estabelecimentos de saúde e orçamento público passaram a ser disponibilizados não apenas para pesquisadores, mas também para toda a população. Esse modelo de dados abertos é singular por sua inovação, variedade de dados, abrangência temporal e territorial das bases e acesso inclusivo. Informações em formato digital de interesse para a saúde também passaram a ser disponibilizados por diferentes instituições como o Instituto Brasileiro de Geografia e Estatística (IBGE), a Agência Nacional de Saúde Suplementar (ANS), a Agência Nacional de Vigilância Sanitária (Anvisa), além das secretarias de saúde de estados e municípios.

Mesmo antes da disseminação digital, dados administrativos, especialmente sobre mortalidade, eram utilizados no Brasil para a pesquisa em saúde coletiva. Contudo, a facilidade de acesso proporcionada pela adesão de instituições brasileiras ao modelo de dados abertos incentivou esse tipo de uso. Por meio da consulta ao PubMed, identifiquei 461 artigos publicados em CSP que usaram dados administrativos, dos quais 86 abordaram temas relacionados à qualidade. Destacam-se, entre esses, o artigo que é fruto da tese de Claudia Risso de Araujo Lima ^6^. Claudia, que foi membro da equipe do DATASUS, foi uma das responsáveis pela implementação da política de disseminação de informações sobre saúde no Brasil. Publicado em 2009, seu artigo continua sendo referenciado até hoje (96 citações na base Scopus). Sua grande contribuição é realizar uma revisão sobre dimensões de qualidade na avaliação de sistemas de informação em saúde do Brasil.

A publicação de artigos que avaliam a qualidade, sejam dos sistemas de informação, sejam de processos para a vinculação de bases, atende a uma crescente demanda para a adoção de boas práticas na condução e relato de estudos que usam dados secundários ^7^ ^,^ ^8^. Um editorial ^9^ e um artigo de perspectivas ^10^ reforçam a política editorial de CSP de valorização do uso responsável de bases administrativas na pesquisa.

CSP também publicou quatro artigos metodológicos que apresentam rotinas computacionais para o processamento de bases de dados. Três soluções foram voltadas para o relacionamento de dados (*record linkage*) ^11^ ^,^ ^12^ ^,^ ^13^, enquanto a última, o pacote Microdatasus ^14^, otimiza o *download* e o pré-processamento de microdados disponibilizados pelo DATASUS. O software Reclink foi publicado em 2000, como software livre de código fechado ^11^. A nova versão OpenReclink foi publicada em 2015, já com código aberto ^12^. Também são de código aberto o EPPD ^13^ e o Microdatasus ^14^, atendendo à política editorial de CSP de adesão à ciência aberta ^15^.

Nesses 40 anos, houve uma expansão acelerada das tecnologias de informação. Avanços na capacidade de captação, processamento, armazenamento, transmissão e análise de dados foram ocorrendo sucessivamente, com avanços incrementais em cada área, estimulando o avanço nas demais. Atualmente, é possível processar grandes quantidades de informações em tempo real. Dados não estruturados que apresentam diferentes formatos, como textos em documentos ou redes sociais, imagens e saídas de sensores, são novas fontes para uso secundário em pesquisas. Adicionalmente, ocorre a introdução na pesquisa em saúde de técnicas desenvolvidas pela Ciência da Informação como a mineração de dados, o aprendizado de máquinas e os modelos de linguagem ampla (*large language models* - LLM). Essas inovações levaram à criação de um novo campo disciplinar, denominado Ciência de Dados Populacionais ^16^ ^,^ ^17^, que, por meio de organização, integração, vinculação e análise de dados individuais e contextuais, objetiva gerar evidências ao nível populacional com valor para a sociedade. Os artigos sobre o desenvolvimento ou aplicação de técnicas de *record linkage* são publicados em CSP desde os anos 2000. Recentemente, com a maior disseminação na Saúde Coletiva das técnicas desenvolvidas pela Ciência da Informação, foram publicados artigos usando mineração de dados, texto e aprendizado de máquina.

Além das questões técnicas, a Ciência de Dados Populacionais busca modelos de gestão de acesso à informação que equilibrem o direito à proteção de informações pessoais com os benefícios potenciais da utilização das bases administrativas na pesquisa, tema tratado em mais de um artigo publicado em CSP ^18^ ^,^ ^19^ ^,^ ^20^.

Ao longo de 40 anos, CSP publicou artigos abordando os principais tópicos da Ciência de Dados Populacionais, valorizando as boas práticas no emprego de dados secundários em pesquisas de interesse para a sociedade. Coerentemente com sua missão, mostrou-se um veículo crucial para a circulação de ideias e métodos desse campo.

# Translation: De los datos secundarios a la Ciencia de Datos Poblacional: recordando los 40 años de producción científica en CSP

**DOI:** 10.1590/0102-311XES087624

**Authors:** Cláudia Medina Coeli

**Affiliations:** 1 Instituto de Estudos em Saúde Coletiva, Universidade Federal do Rio de Janeiro, Rio de Janeiro, Brasil.

Me complació mucho la invitación a escribir este editorial. Esta es una gran oportunidad para celebrar junto a Marilia Sá Carvalho, a Luciana Dias de Lima, a Luciana Correia Alves y a toda la comunidad CSP el 40º aniversario de este importante proyecto editorial, en el que tuve el honor de participar durante nueve años como Coeditora en Jefe. Teniendo como foco principal estudios sobre el desarrollo de técnicas y el uso de bases de datos secundarias, revisar la producción científica en esta temática en CSP me permitió recordar artículos fundamentales en mi formación y desarrollo de mis proyectos de investigación.

La primera edición de CSP se publicó en 1985. En el contexto internacional, cobraba impulso la venta de computadoras personales (PC) ^1^, seguida de la apertura al público del acceso a la World Wide Web (WWW) a principios de los 1990 ^2^. Estos avances resultaron en la popularización de las tecnologías de la información.

Las bases administrativas comenzaron a utilizarse como fuentes de datos secundarias en la investigación en Salud Pública ^3^. En los años 1990, y en la primera década de los 2000, se implementaron Centros de Datos en Australia, en Canadá y en Reino Unido. En estas organizaciones, las bases administrativas están continuamente vinculadas, y los conjuntos de datos anonimizados resultantes pueden ser accedidos por los investigadores para desarrollar sus proyectos ^4^.

En 1991 Brasil creó el Departamento de Informática del Sistema Único de Salud (DATASUS) ^5^, lo que implicó un gran aporte a la accesibilidad en las bases administrativas brasileñas. Pero adoptó un modelo para la difusión de datos distinto del Centro de Datos mencionado anteriormente. En el modelo estaban habilitadas dos modalidades de acceso. La primera se daba por un tabulador en línea que permitía crear tablas de los principales Sistemas de Información Sanitaria nacionales. La segunda consistía en la difusión de microdatos no identificados. Al principio, las bases se distribuían en discos compactos (CD) mensuales y, posteriormente, se pusieron a disposición para descarga en línea. Los datos de nacimientos, defunciones, enfermedades y enfermedades de declaración obligatoria, atención primaria, atención ambulatoria y hospitalaria, centros de salud y presupuesto público comenzaron a ponerse a disposición no solo a los investigadores, sino también a toda la población. Ese modelo de Datos Abiertos es innovador por la variedad de datos, la cobertura temporal y territorial de bases de datos y el acceso inclusivo. La información en formato digital en torno a la salud también fue puesta a disposición por varias instituciones como el Instituto Brasileño de Geografía y Estadística (IBGE), la Agencia Nacional de Salud Suplementaria (ANS), la Agencia Nacional de Vigilancia Sanitaria (Anvisa), además de los departamentos de salud de estados y municipios.

Incluso antes de la difusión digital, los datos administrativos, especialmente sobre mortalidad, se utilizaban en Brasil en los estudios de Salud Pública. Sin embargo, el acceso proporcionado por la adhesión de las instituciones brasileñas al modelo de datos abiertos fomentó este tipo de uso. En una búsqueda en PubMed, encontré 461 artículos publicados en CSP que utilizaban datos administrativos; de los cuales 86 abordaban temas relacionados con la calidad. Entre estos últimos, se destaca el artículo que es resultado de la tesis de Claudia Risso de Araujo Lima ^6^. Claudia, ex miembro del equipo de DATASUS, fue una de las responsables de implementar la política de difusión de información de salud en Brasil. Su artículo publicado en 2009 sigue siendo un referente (96 citas en la base de datos Scopus). Su gran aporte fue realizar una revisión de las dimensiones de calidad en la evaluación de los sistemas de información de salud en Brasil.

La publicación de artículos que evalúan la calidad de sistemas de información o de procesos de vinculación de bases de datos responde a una creciente demanda de adopción de buenas prácticas en la realización y reporte de estudios que utilizan datos secundarios ^7^ ^,^ ^8^. Un editorial ^9^ y un artículo de perspectivas ^10^ refuerzan la política editorial de CSP de valorar el uso responsable de las bases administrativas en la investigación.

CSP también publicó cuatro artículos metodológicos que presentan rutinas computacionales para el procesamiento de bases de datos. Tres soluciones estaban dirigidas a la vinculación de registros ^11^ ^,^ ^12^ ^,^ ^13^, mientras que la última, el paquete Microdatasus ^14^, optimiza la descarga y el preprocesamiento de los microdatos puestos a disposición por DATASUS. El software Reclink se lanzó en 2000 como software libre, pero de código cerrado ^11^. La nueva versión de OpenReclink se publicó en 2015, ya con código abierto ^12^. EPPD ^13^ y Microdatasus ^14^ también son de código abierto en cumplimiento con la política editorial de CSP de adhesión a la ciencia abierta ^15^.

En estos 40 años ha habido una creciente expansión de las tecnologías de la información. Los avances en la capacidad de capturar, procesar, almacenar, transmitir y analizar datos se produjeron sucesivamente, con avances en cada área estimulando el progreso en las demás. Actualmente, es posible procesar grandes cantidades de información en tiempo real. Los datos no estructurados que presentan diferentes formatos, como textos en documentos o redes sociales, imágenes y salidas de sensores, son nuevas fuentes de uso secundario en la investigación. Además, existe la introducción en la investigación en salud de técnicas desarrolladas por la Ciencia de la Información como la minería de datos, el aprendizaje automático y los modelos lingüísticos de gran tamaño (*large language models* -LLM). Estas innovaciones posibilitaron la creación de un nuevo campo, la Ciencia de Datos Poblacionales ^16^ ^,^ ^17^, que mediante la organización, integración, vinculación y análisis de datos individuales y contextuales pretende generar evidencia a nivel poblacional con valor para la sociedad. Los artículos sobre el desarrollo o la aplicación de técnicas de vinculación de registros se han publicado en CSP desde los años 2000. Con la recién difusión en Salud Pública de las técnicas desarrolladas por la Ciencia de la Información, se han publicado artículos utilizando minería de datos, texto y aprendizaje automático.

Además de las cuestiones técnicas, la Ciencia de Datos Poblacionales busca modelos de gestión de acceso a la información que equilibren el derecho a la protección de la información personal con los potenciales beneficios para la sociedad del uso de bases administrativas en la investigación, un tema abordado en más de un artículo ya publicado en CSP ^18^ ^,^ ^19^ ^,^ ^20^.

A lo largo de estos 40 años CSP ha publicado artículos que abordan los principales temas de la Ciencia de Datos Poblacionales, valorando las buenas prácticas con el uso de datos secundarios en la investigación de interés para la sociedad. En consonancia con su misión, demostró ser una herramienta esencial en la circulación de ideas y métodos en este campo.
